# Application of the Five-Step Phase-Shifting Method in Reflective Ghost Imaging for Efficient Phase Reconstruction

**DOI:** 10.3390/s24020320

**Published:** 2024-01-05

**Authors:** Ziyan Chen, Jing Cheng, Heng Wu

**Affiliations:** 1 Guangdong Provincial Key Laboratory of Cyber-Physical System, School of Automation, Guangdong University of Technology, Guangzhou 510006, China; zychen@gdut.edu.cn; 2 School of Computer, Guangdong University of Technology, Guangzhou 510006, China; 3 School of Physics, South China University of Technology, Guangzhou 510641, China; phjcheng@scut.edu.cn

**Keywords:** Reflective Ghost Imaging, phase reconstruction, Five-Step Phase-Shifting (FSPS) method, signal processing

## Abstract

The conventional approach to phase reconstruction in Reflective Ghost Imaging (RGI) typically involves the introduction of three reference screens into the reference path, deeming the Fourier transform step indispensable. However, this method introduces complexity to the system and raises concerns regarding potential errors in phase retrieval. In response to these challenges, we advocate for adopting the Five-Step Phase-Shifting (FSPS) method in the RGI system. This method presents two key advantages over traditional approaches: (1) It streamlines the phase reconstruction process by eliminating the requirement for a Fourier inverse transform. (2) It avoids the need to insert objects into the reference optical path, simplifying the computation of reference optical path intensity and enabling seamless application to Computational Ghost Imaging (CGI), overcoming the constraints of Dual-Arm Ghost Imaging (DAGI). We substantiate the theoretical proposition through numerical simulations involving two intricate objects. Furthermore, our discussion delves into exploring the influence of varying reflective angles on the phase reconstruction performance.

## 1. Introduction

Ghost Imaging (GI) has garnered substantial attention in recent years due to its nonlocal probing and single-pixel detecting properties. It reconstructs the image of an unknown object by measuring the intensity correlation between test and reference path detectors [1,2,3,4,5,6,7,8,9,10,11,12,13,14,15,16,17,18]. Recent years have witnessed significant advancements in signal processing through the application of deep learning techniques [19]. These methods leverage the capabilities of neural networks to comprehend intricate patterns and relationships, providing a potent tool for further enhancing the capabilities of ghost imaging across various applications [20,21]. On the one hand, GI technology excels in low-light imaging, demonstrating proficiency in producing clear images from weak light signals. This capability lends itself to a wide range of applications, including remote sensing [22,23], watermark technology [24], medical imaging [25], and cryptography [26,27]. On the other hand, GI can handle not only transmissive objects [1,16,28,29,30,31,32,33,34,35,36,37,38,39] but also reflective ones [40,41,42,43,44,45,46,47,48]. Numerous reports indicate the successful reconstruction of both amplitude and phase distribution of transmissive objects [16,28,29,30,31,32,33,34,35,36,37,38,39] in Transmissive Ghost Imaging (TGI).

Obtaining phase information about objects is crucial in various fields, especially in optics [49], electronics [50], communications [51], medicine [52], and other scientific and engineering domains. For instance, in medicine, phase information plays a vital role in various imaging techniques, including magnetic resonance imaging and optical coherence tomography. Analyzing the phase information allows doctors to gain insights into tissue structures, vascular distribution, and abnormalities for diagnosis and treatment planning.

The commonly employed GI technique is used to determine the reflectivity of a reflective object, but it leaves the phase distribution of the reflection coefficient unknown. In recent years, efforts have been made to reconstruct the phase distribution information in Reflective Ghost Imaging (RGI) [46]. In [46], Chen successfully reconstructed the reflected object’s phase distribution by inserting reference screens into the reference path and employing the Three-Step Phase-Shifting (TSPS) method. However, the inclusion of a Fourier transform step in this method is necessary for reconstructing phase information, potentially introducing a phase retrieval error. Moreover, the incorporation of a reference screen in the reference arm restricts the applicability of this approach to computational ghost imaging (CGI) [3,4,5,6,7] and confines its use to Dual-Arm Ghost Imaging (DAGI) applications.

In a recent contribution, Chen introduced an innovative approach that utilizes the Five-Step Phase-Shifting (FSPS) method for reconstructing the phase distribution of a complex transmissive object in TGI [34]. This method offers distinct advantages by eliminating the need for iterative algorithms [35,36,37], a complex GI system [30,53], entangled photon pairs [39], or Fourier transform steps [28,29,33,46,54]. Additionally, the FSPS method transcends the limitations of DAGI and can be seamlessly applied to CGI.

Building upon these advancements, we spontaneously embarked on exploring the potential application of the novel FSPS method to RGI for obtaining the phase distribution of reflective objects. While the FSPS method was initially proposed in the context of scenarios involving TGI, our detailed theoretical derivations focused on RGI scenarios, revealing its direct applicability to enhance phase reconstruction in RGI.

In this paper, we take the initial step of providing a detailed theoretical derivation of the FSPS method in RGI. It is worth noting that, although we utilize a DAGI framework for clarity, the current framework can be seamlessly adapted for CGI as long as the reference arm contains no objects. Subsequently, we investigate the impact of various incident and reflective angles on this phase reconstruction method through numerical simulations. Our deliberate selection of the most suitable incident and reflective angles aims to showcase the robustness and efficiency of the proposed phase retrieval approach. In summary, our work bridges the gap between the FSPS method initially proposed for TGI and its direct application to enhance phase reconstruction in RGI. The adaptability of the FSPS method and its robust performance in diverse scenarios highlight its potential as a valuable tool in advancing RGI techniques.

## 2. Model and Theory

**Figure 1:**
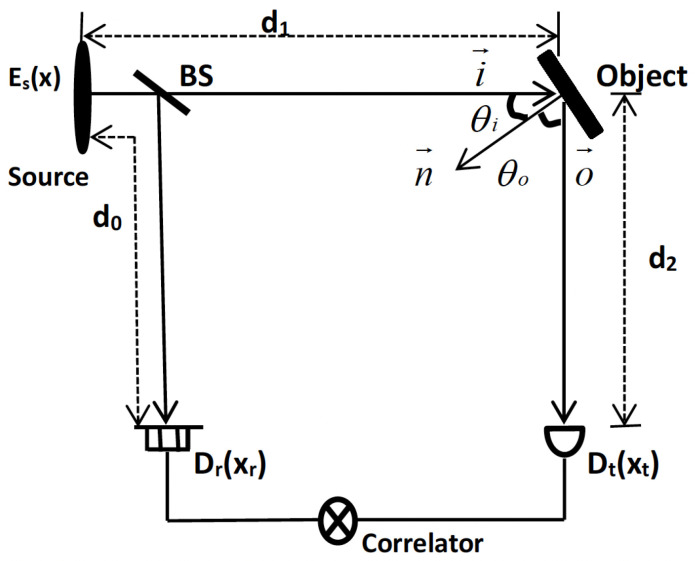
Geometry of our generalized RGI system: The source field $E_{s}\left(x\right)$ undergoes beam splitting by the non-polarized BS, where *x*, *u*, $x_{t}$ , and $x_{r}$ denote the positions at the source plane, unknown object plane, test detector plane, and reference detector plane, respectively. $\vec{n}$ , $\vec{i}$ , and $\vec{o}$ represent unit vectors pointing in the direction of the average surface normal, incident light, and the detection plane, respectively. $\theta_{i}$ and $\theta_{o}$ are the incident angle and the reflective angle, respectively. $D_{t}$ and $D_{r}$ denote the detectors in the test and reference paths, respectively. The term “correlator” refers to any device capable of processing signals, such as a computer.

We present our generalized Reflective Ghost Imaging (RGI) scheme in Figure 1. Notably, it closely resembles the conventional RGI system. The classical source undergoes division into two beams facilitated by the beam splitter (BS). Subsequently, these two beams traverse distinct paths: the reference path and the test path. Within the test path, an unknown reflective object is introduced, with $d_{2}$ denoting the distance from the object to the test detector $D_{t}$ , and $d_{1}$ representing the distance from the source to the object. Simultaneously, the reference path remains unaffected by the object and incorporates a high-resolution sensor $D_{r}$ . The distance between the source and the detector $D_{r}$ is designated as $d_{0}$ .

Theoretically, the reflective object can be described by the speckle model, with its random surface height assumed to follow Gaussian statistics. As discussed in Refs. [44,55], the relationship between the reflected fields $E_{o}$ at the surface of the object and the incident field $E_{i}$ is expressed as follows:

(1) $$\begin{array}{c} E_{o}\left(u\right)=E_{i}\left(u\right)r\left(u\right)\exp\left(j\phi \left(u\right)\right). \end{array}$$

Here, r(u) represents the reflection coefficient of the object, and ϕ(u) signifies the phase delay proportional to the random surface height of the object h(u) :

(2) $$\begin{array}{c} \phi \left(u\right)=k\left(-\vec{i}\cdot \vec{n}+\vec{o}\cdot \vec{n}\right)h\left(u\right) \end{array}$$

where *k* represents the wave number of the incident light, and the dot product $\vec{o}\cdot \vec{n}$ is the cosine of the angle between the unit vectors $\vec{o}$ and $\vec{n}$ .

Using Equation (1), the field $E_{t}\left(x_{t}\right)$ at the test detector $D_{t}$ can be calculated as [56]

(3) $$\begin{array}{ccc} E_{t}\left(x_{t}\right) & = & \frac{-1}{\lambda \sqrt{d_{1}d_{2}}}\int dudx_{1}E_{s}\left(x_{1}\right)r\left(u\right)\exp\left[i\phi \left(u\right)\right]\\ & & \times \exp\left[-\frac{i\pi }{\lambda d_{1}}{\left(x_{1}-u\cos\theta_{i}\right)}^{2}\right]\\ & & \times \exp\left[-\frac{i\pi }{\lambda d_{2}}{\left(u\cos\theta_{o}-x_{t}\right)}^{2}\right] \end{array}$$

where $\theta_{i}$ and $\theta_{o}$ represent the incident angle and reflective angle, respectively. Similarly, we obtain the field $E_{r}\left(x_{r}\right)$ as follows:

(4) $$\begin{array}{ccc} E_{r}\left(x_{r}\right) & = & \frac{1}{\sqrt{i\lambda d_{0}}}\int dx_{2}E_{s}\left(x_{2}\right)\exp\left[-\frac{i\pi }{\lambda d_{0}}{\left(x_{2}-x_{r}\right)}^{2}\right]. \end{array}$$

Combined with classical optical coherent theory [56] and the notations in [44], and considering that a complex circular Gaussian random process can model the field fluctuations of a classical light source with zero mean [57], we have

(5) $$\begin{array}{ccc} \langle E_{s}\left(x_{1}\right)E_{s}^{*}\left(x_{1}^{\prime }\right)E_{s}\left(x_{2}\right)E_{s}^{*}\left(x_{2}^{\prime }\right)\rangle & = & \langle E_{s}\left(x_{1}\right)E_{s}^{*}\left(x_{2}^{\prime }\right)\rangle \langle E_{s}^{*}\left(x_{1}^{\prime }\right)E_{s}\left(x_{2}\right)\rangle \\ & & +\langle E_{s}\left(x_{1}\right)E_{s}^{*}\left(x_{1}^{\prime }\right)\rangle \langle E_{s}\left(x_{2}\right)E_{s}^{*}\left(x_{2}^{\prime }\right)\rangle \end{array}$$

where 〈〉 denotes the ensemble average, the correlation of intensity fluctuations between the test and reference detectors can be calculated as follows:

(6) $$\begin{array}{ccc} G(x_{t},x_{r}) & = & \langle E_{r}\left(x_{r}\right)E_{r}^{*}\left(x_{r}\right)E_{t}\left(x_{t}\right)E_{t}^{*}\left(x_{t}\right)\rangle -\langle E_{t}\left(x_{t}\right)E_{t}^{*}\left(x_{t}\right)\rangle \langle E_{r}\left(x_{r}\right)E_{r}^{*}\left(x_{r}\right)\rangle \\ & = & \frac{1}{\lambda^{3}d_{0}d_{1}d_{2}}\int dudu^{\prime }\langle r\left(u\right)r^{*}\left(u^{\prime }\right)\exp\left[i\phi \left(u\right)-i\phi \left(u^{\prime }\right)\right]\rangle \\ & & \times \int dx_{1}dx_{1}^{\prime }dx_{2}dx_{2}^{\prime }\langle E_{s}^{*}\left(x_{1}^{\prime }\right)E_{s}\left(x_{2}\right)\rangle \langle E_{s}^{*}\left(x_{2}^{\prime }\right)E_{s}\left(x_{1}\right)\rangle \\ & & \times \exp\left\{\frac{i\pi }{\lambda d_{1}}\left[{\left(x_{1}^{\prime }-u^{\prime }\cos\theta_{i}\right)}^{2}-{\left(x_{1}-u\cos\theta_{i}\right)}^{2}\right]\right\}\rangle \\ & & \times \exp\left\{\frac{i\pi }{\lambda d_{2}}\left[{\left(u^{\prime }\cos\theta_{o}-x_{t}\right)}^{2}-{\left(u\cos\theta_{o}-x_{t}\right)}^{2}\right]\right\}\\ & & \times \exp\left\{\frac{i\pi }{\lambda d_{0}}\left[{\left(x_{2}^{\prime }-x_{r}\right)}^{2}-{\left(x_{2}-x_{r}\right)}^{2}\right]\right\}. \end{array}$$

In TGI, only one stochastic fluctuation originates from the source field. However, in RGI, the scenario is more intricate as it involves two independent types of stochastic fluctuations: one from the source and the other from the object itself [46]. Let us define the stochastic fluctuation from the object as $R\left(u,u^{\prime }\right)=\langle r\left(u\right)r^{*}\left(u^{\prime }\right)\exp\left[i\phi \left(u\right)-i\phi \left(u^{\prime }\right)\right]\rangle$ , and it is independent of the source fluctuation. Mathematically, $R\left(u,u^{\prime }\right)=r\left(u\right)r^{*}\left(u^{\prime }\right)\exp\left\{-\sigma_{\phi }^{2}\left[1-\exp\left(-{\left(u-u^{\prime }\right)}^{2}/\ell_{c}^{2}\right)\right]\right\}$ [58], where r(u) represents the reflection coefficient of the object, $\ell_{c}$ is the surface correlation length, and $\sigma_{\phi }^{2}$ is the variance of the phase related to the variance of the surface height $\sigma_{h}^{2}$ ,

(7) $$\begin{array}{c} \sigma_{\phi }^{2}=\left[k\left(-\vec{i}\cdot \vec{n}+\vec{o}\cdot \vec{n}\right)\right]\sigma_{h}^{2}. \end{array}$$

Suppose the source is entirely incoherent; we have

(8) $$\begin{array}{ccc} & & \langle E_{s}^{*}\left(x_{2}^{\prime }\right)E_{s}\left(x_{1}\right)\rangle =f\left(x_{1}\right)\mathrm{rect}\left(\frac{x_{1}}{s_{e}}\right)\delta \left(x_{1}-x_{2}^{\prime }\right)\\ & & \langle E_{s}^{*}\left(x_{1}^{\prime }\right)E_{s}\left(x_{2}\right)\rangle =f\left(x_{2}\right)\mathrm{rect}\left(\frac{x_{2}}{s_{e}}\right)\delta \left(x_{2}-x_{1}^{\prime }\right) \end{array}$$

in which $f(x_{1})$ represents the intensity distribution of the source, rect(x) is the rectangular function, and $s_{e}$ denotes the size of the incoherent source.

In implementing GI, we set $d_{0}=d_{1}$ , and position a point detector in the test path. The expression for the GI formula can be articulated as follows:

(9) $$\begin{array}{ccc} I(x_{r}) & = & G(x_{t}=0,x_{r})\\ & = & \frac{1}{\lambda^{3}d_{0}^{2}d_{2}}\int dx_{1}dx_{2}dudu^{\prime }R\left(u,u^{\prime }\right)f\left(x_{1}\right)f\left(x_{2}\right)\mathrm{rect}\left(\frac{x_{1}}{s_{e}}\right)\mathrm{rect}\left(\frac{x_{2}}{s_{e}}\right)\\ & & \times \exp\left\{\frac{i\pi }{\lambda d_{0}}\left[{\left(x_{2}-u^{\prime }\cos\theta_{i}\right)}^{2}-{\left(x_{1}-u\cos\theta_{i}\right)}^{2}+{\left(x_{1}-x_{r}\right)}^{2}-{\left(x_{2}-x_{r}\right)}^{2}\right]\right\}\\ & & \times \exp\left[\frac{i\pi \cos^{2}\theta_{o}}{\lambda d_{2}}\left({u^{\prime }}^{2}-u^{2}\right)\right]. \end{array}$$

To simplify the formula, we introduce the following symbols:

$$\begin{array}{c} k_{0}=\frac{i\pi }{\lambda d_{0}},k_{2}=\frac{i\pi \cos^{2}\theta_{o}}{\lambda d_{2}\cos^{2}\theta_{i}},A=\frac{1}{\lambda^{3}d_{0}^{2}d_{2}}. \end{array}$$

Moreover, we substitute variables with $y=u\cos\theta_{i}$ and $y^{\prime }=u^{\prime }\cos\theta_{i}$ . Assuming the incoherent source size is sufficiently large, the formula can be presented as:

(10) $$\begin{array}{ccc} I(x_{r}) & = & \frac{A}{\cos^{2}\theta_{i}}\int dx_{1}dx_{2}f\left(x_{1}\right)f\left(x_{2}\right)\\ & & \times dydy^{\prime }R\left(\frac{y}{\cos\theta_{i}},\frac{y^{\prime }}{\cos\theta_{i}}\right)\exp\left[k_{2}\left({y^{\prime }}^{2}-y^{2}\right)\right]\\ & & \times \exp\left\{k_{0}\left[{\left(x_{2}-y^{\prime }\right)}^{2}-{\left(x_{1}-y\right)}^{2}\right]\right\}\\ & & \times \exp\left\{k_{0}\left[{\left(x_{1}-x_{r}\right)}^{2}-{\left(x_{2}-x_{r}\right)}^{2}\right]\right\}\\ & = & \frac{A}{\cos^{2}\theta_{i}}\int dydy^{\prime }R\left(\frac{y}{\cos\theta_{i}},\frac{y^{\prime }}{\cos\theta_{i}}\right)\\ & & \times \exp\left[\left(k_{0}+k_{2}\right)\left({y^{\prime }}^{2}-y^{2}\right)\right]\\ & & \times dx_{1}f\left(x_{1}\right)\exp\left[2k_{0}x_{1}\left(y-x_{r}\right)\right]\\ & & \times dx_{2}f\left(x_{2}\right)\exp\left[2k_{0}x_{2}\left(x_{r}-y^{\prime }\right)\right]. \end{array}$$

Especially, when $\ell_{c}$ is significantly large, we obtain $R\left(u,u^{\prime }\right)=r\left(u\right)r^{*}\left(u^{\prime }\right)$ , resulting in the GI pattern as:

(11a) $$\begin{array}{ccc} I(x_{r}) & = & \frac{A}{\cos^{2}\theta_{i}}{\left|\int dy\;r\left(\frac{y}{\cos\theta_{i}}\right)\exp\left[-\left(k_{0}+k_{2}\right)y^{2}\right]dx_{1}f\left(x_{1}\right)\exp\left[2k_{0}x_{1}\left(y-x_{r}\right)\right]\right|}^{2}\\ & = & \frac{A}{\cos^{2}\theta_{i}}{\left|\int dyF\left(\frac{x_{r}-y}{i\pi /k_{0}}\right)r\left(\frac{y}{\cos\theta_{i}}\right)\exp\left[-\left(k_{0}+k_{2}\right)y^{2}\right]\right|}^{2}\\ & = & \frac{A}{\cos^{2}\theta_{i}}{\left|F\left(\frac{x_{r}}{i\pi /k_{0}}\right)⊗\left(r\left(\frac{x_{r}}{\cos\theta_{i}}\right)\exp\left[-\left(k_{0}+k_{2}\right)x_{r}^{2}\right]\right)\right|}^{2} \end{array}$$

(11b) $$\begin{array}{ccc} & = & \frac{1}{\lambda^{3}d_{0}^{2}d_{2}\cos^{2}\theta_{i}}{\left|F\left(\frac{x_{r}}{\lambda d_{0}}\right)⊗\left(r\left(\frac{x_{r}}{\cos\theta_{i}}\right)\exp\left[-\frac{i\pi }{\lambda }\left(\frac{1}{d_{0}}+\frac{1}{d_{2}}\times \frac{\cos^{2}\theta_{o}}{\cos^{2}\theta_{i}}\right)x_{r}^{2}\right]\right)\right|}^{2} \end{array}$$

in which ⊗ denotes convolution, and F(.) represents the Fourier transform of f(.) . It is evident that Equation (11b) bears a strong resemblance to Equation (7) introduced in [34], suggesting that the FSPS method, originally proposed for TGI in [34], could be extended to RGI.

For simplicity, we substitute the object $r_{e}\left(x_{r}\right)$ for $r\left(\frac{x_{r}}{\cos\theta_{i}}\right)\exp\left[-\frac{i\pi }{\lambda }\left(\frac{1}{d_{0}}+\frac{1}{d_{2}}\times \frac{\cos^{2}\theta_{o}}{\cos^{2}\theta_{i}}\right)x_{r}^{2}\right]$ . Consequently, we obtain:

(12) $$\begin{array}{c} r_{e}\left(x_{r}\right)=r\left(\frac{x_{r}}{\cos\theta_{i}}\right)\exp\left[-\frac{i\pi }{\lambda }\left(\frac{1}{d_{0}}+\frac{1}{d_{2}}\times \frac{\cos^{2}\theta_{o}}{\cos^{2}\theta_{i}}\right)x_{r}^{2}\right]. \end{array}$$

By acquiring the phase and amplitude details of the object $r_{e}\left(x_{r}\right)$ , it becomes evident that we can subsequently reconstruct the phase and amplitude distributions of $r(x_{r})$ using Equation (12).

Utilizing Equation (12), we can represent Equation (11b) as follows:

(13) $$\begin{array}{c} I\left(x_{r}\right)=\frac{1}{\lambda^{3}d_{0}^{2}d_{2}\cos^{2}\theta_{i}}{\left|F\left(\frac{x_{r}}{\lambda d_{0}}\right)⊗r_{e}\left(x_{r}\right)\right|}^{2}. \end{array}$$

It is evident that Equation (13) is identical to Equation (9) derived in [34], reinforcing the idea that the FSPS method proposed in [34] for TGI can be directly applied to RGI. Thus, with the FSPS method [34], one can directly obtain:

(14a) $$\begin{array}{ccc} H(x_{r}) & = & G_{1}I_{1}\left(x_{r}\right)+G_{2}I_{2}\left(x_{r}\right)+G_{3}I_{3}\left(x_{r}\right)+G_{4}I_{4}\left(x_{r}\right)+G_{5}I_{5}\left(x_{r}\right) \end{array}$$

(14b) $$\begin{array}{ccc} & = & r_{e}\left(x_{r}-\varepsilon \lambda d_{0}\right)r_{e}^{*}\left(x_{r}+\varepsilon \lambda d_{0}\right) \end{array}$$

where $G_{1}=\frac{\left(2\sqrt{2}-2\right)\left(1+i\right)}{2\sqrt{2}-4}$ , $G_{2}=\frac{\left[-2\sqrt{2}+i\left(2\sqrt{2}-4\right)\right]}{2\sqrt{2}-4}$ , $G_{3}=\frac{4}{2\sqrt{2}-4}$ , $G_{4}=\frac{\left[-2\sqrt{2}-i\left(2\sqrt{2}-4\right)\right]}{2\sqrt{2}-4}$ , $G_{5}=\frac{\left(2\sqrt{2}-2\right)\left(1-i\right)}{2\sqrt{2}-4}$ , and $I_{m}\left(x_{r}\right)=\frac{1}{\lambda^{3}d_{0}^{2}d_{2}\cos^{2}\theta_{i}}{\left|r_{e}\left(x_{r}\right)+u_{m}r_{e}\left(x_{r}-\varepsilon \lambda d_{0}\right)+u_{m}^{*}r_{e}\left(x_{r}+\varepsilon \lambda d_{0}\right)\right|}^{2}$ , with m=1,2,3,4,5 and $u_{1}=\frac{1}{2}$ , $u_{2}=\frac{1+i}{2\sqrt{2}}$ , $u_{3}=\frac{i}{2}$ , $u_{4}=\frac{-1+i}{2\sqrt{2}}$ , $u_{5}=-\frac{1}{2}$ . Here, ε denotes a real constant.

Then we have

(15) $$\begin{array}{c} \Phi_{H}\left(x_{r}\right)=\Phi_{r_{e}}\left(x_{r}-\varepsilon \lambda d_{0}\right)-\Phi_{r_{e}}\left(x_{r}+\varepsilon \lambda d_{0}\right) \end{array}$$

where $\Phi_{H}\left(x_{r}\right)$ represents the phase of $H(x_{r})$ , and $\Phi_{r_{e}}\left(x_{r}\right)$ represents the phase of $r_{e}\left(x_{r}\right)$ , the quantitative reconstruction of the phase of $r_{e}\left(x_{r}\right)$ from the phase information of $H(x_{r})$ can be achieved using Equation (15). The specific steps are as follows: (1) Assume that the phase at $x_{r}=0$ is zero, i.e., $\Phi_{r_{e}}\left(0\right)=0$ . (2) By applying Equation (15), one can determine the values of $\Phi_{r_{e}}\left(0\right)$ , $\pm \Phi_{r_{e}}\left(2\varepsilon \lambda d_{0}\right)$ , $\pm \Phi_{r_{e}}\left(4\varepsilon \lambda d_{0}\right)$ , and so forth.

Upon the successful reconstruction of the phase information of $r_{e}\left(x_{r}\right)$ from the phase information of $H(x_{r})$ , the phase information of $r(\frac{x_{r}}{\cos\theta_{i}})$ can be obtained using Equation (12):

(16) $$\begin{array}{c} \Phi_{r}\left(\frac{x_{r}}{\cos\theta_{i}}\right)=\Phi_{r_{e}}\left(x_{r}\right)+\frac{\pi }{\lambda }\left(\frac{1}{d_{0}}+\frac{1}{d_{2}}\times \frac{\cos^{2}\theta_{o}}{\cos^{2}\theta_{i}}\right)x_{r}^{2} \end{array}$$

The amplitude of $r_{e}\left(x_{r}\right)$ can be determined using the standard RGI scheme without the designed shaped source. By applying Equation (12), we can directly infer that the amplitude of $r_{e}\left(x_{r}\right)$ is equivalent to the amplitude information of $r\left(\frac{x_{r}}{\cos\theta_{i}}\right)$ . Therefore,

(17) $$\begin{array}{c} \left|r\left(\frac{x_{r}}{\cos\theta_{i}}\right)\right|=\left|r_{e}\left(x_{r}\right)\right|. \end{array}$$

With Equations (16) and (17), we can ascertain the amplitude and phase of $r\left(\frac{x_{r}}{\cos\theta_{i}}\right)$ . Subsequently, the phase and amplitude information of $r(x_{r})$ can be directly reconstructed from $r\left(\frac{x_{r}}{\cos\theta_{i}}\right)$ through coordinate transformation. Assuming a zero phase at $r_{e}\left(x_{r}\right)=0$ denoted as $\phi_{r_{e}}\left(0\right)=0$ , the reconstructed phase exhibits a constant value difference from the actual phase. Nevertheless, the absolute phase holds little significance, given that the relative phase distribution remains unchanged.

## 3. Numerical Simulations

In the subsequent discussions, we verify the effectiveness of our RGI scheme by employing two types of complex reflective objects: a Reflective Double-Slit (RDS) and a Reflective Double-Slit Gaussian Phase Plate (RDSGPP). In our simulations, we configure the transverse size of the source as $D_{s}=10\;\mathrm{mm}$ , the wavelength as λ=628nm , and the distances $d_{1}=400\;\mathrm{mm}$ and $d_{2}=200\;\mathrm{mm}$ . The Charge-Coupled Device (CCD) resolution is $\Delta_{x_{r}}=8.3\;\mu m$ , the sample number is M=320 , and ε is specified as $33.041\;m^{-1}$ . Here, let us elaborate on why the value of ε is chosen as $33.041\;m^{-1}$ . The reason can be traced back to Equation (15). The discretized form of Equation (15) is expressed as follows:

(18) $$\begin{array}{ccc} \Phi_{H}\left(M\Delta x_{r}\right) & = & \Phi_{r_{e}}\left(M\Delta x_{r}-\Delta x_{r}\right)-\Phi_{r_{e}}\left(M\Delta x_{r}+\Delta x_{r}\right)\\ & = & \Phi_{r_{e}}\left[\left(M-1\right)\Delta x_{r}\right]-\Phi_{r_{e}}\left[\left(M+1\right)\Delta x_{r}\right] \end{array}$$

where $\Delta x_{r}=\varepsilon \lambda d_{0}$ , and $\Delta x_{r}$ represents the pixel size of the CCD ( $\Delta x_{r}=8.3\;\mu m$ ). Additionally, $x_{r}$ must be divisible by $\Delta x_{r}$ (i.e., $x_{r}/\Delta x_{r}=M$ , where *M* is an integer, and here we set M=320 ). This discrete representation proves to be more convenient for subsequent signal processing in our simulated experiments. According to Equation (18), we can extract the values of $\Phi_{r_{e}}$ from $\Phi_{H}$ using a recursive algorithm. Thus, we determine the value of ε as:

$$\varepsilon =\frac{\Delta x_{r}}{\lambda d_{0}}=\frac{8.3\;\mu m}{628\;\mathrm{nm}\times 400\;\mathrm{mm}}=33.041\;m^{-1}.$$

Here, we also outline the steps of our simulation experiment as follows:

1. Perform multidimensional integration on Equation (11a) to calculate the values of $I_{1}\left(x_{r}\right)$ , $I_{2}\left(x_{r}\right)$ , $I_{3}\left(x_{r}\right)$ , $I_{4}\left(x_{r}\right)$ , $I_{5}\left(x_{r}\right)$ , and $I_{0}\left(x_{r}\right)$ . These values can be determined by setting the parameter *u* in the function $f\left(x_{1}\right)=1+ue^{i2\pi \varepsilon x_{1}}+u^{*}e^{-i2\pi \varepsilon x_{1}}$ [34] to $u=\frac{1}{2}$ , $u=\frac{1+i}{2\sqrt{2}}$ , $u=\frac{i}{2}$ , $u=\frac{-1+i}{2\sqrt{2}}$ , $u=-\frac{1}{2}$ , and u=0 . Obtaining the value of $I_{0}\left(x_{r}\right)$ is crucial for extracting the amplitude information of the object: setting *u* to 0 transforms it into a conventional RGI mode. Taking the square root of $I_{0}\left(x_{r}\right)$ yields the amplitude information $\left|r_{e}\left(x_{r}\right)\right|$ of $r_{e}\left(x_{r}\right)$ .

2. Substitute the values of $I_{1}\left(x_{r}\right)$ , $I_{2}\left(x_{r}\right)$ , $I_{3}\left(x_{r}\right)$ , $I_{4}\left(x_{r}\right)$ , and $I_{5}\left(x_{r}\right)$ obtained in the first step into Equation (14a), resulting in $H(x_{r})$ . Then, obtain the phase value $\Phi_{H}\left(x_{r}\right)$ from $H(x_{r})$ . By further applying Equation (18) and using a recursive algorithm, we can extract the phase values of $\Phi_{r_{e}}\left(x_{r}\right)$ .

3. Substitute the obtained values of $\Phi_{r_{e}}\left(x_{r}\right)$ from the second step into Equation (16), successfully obtaining the phase information $\Phi_{r}\left(\frac{x_{r}}{\cos\theta_{i}}\right)$ . Then, applying Equation (17) along with the amplitude $\left|r_{e}\left(x_{r}\right)\right|$ obtained in the first step yields the value of $\left|r\left(\frac{x_{r}}{\cos\theta_{i}}\right)\right|$ .

4. Transform the obtained phase values $\Phi_{r}\left(\frac{x_{r}}{\cos\theta_{i}}\right)$ and amplitude values $\left|r\left(\frac{x_{r}}{\cos\theta_{i}}\right)\right|$ using coordinate transformations to obtain the values of $\Phi_{r}\left(x_{r}\right)$ and $\left|r(x_{r})\right|$ . Thus, the reconstruction of the phase and amplitude of the reflective object is completed.

**Figure 2:**
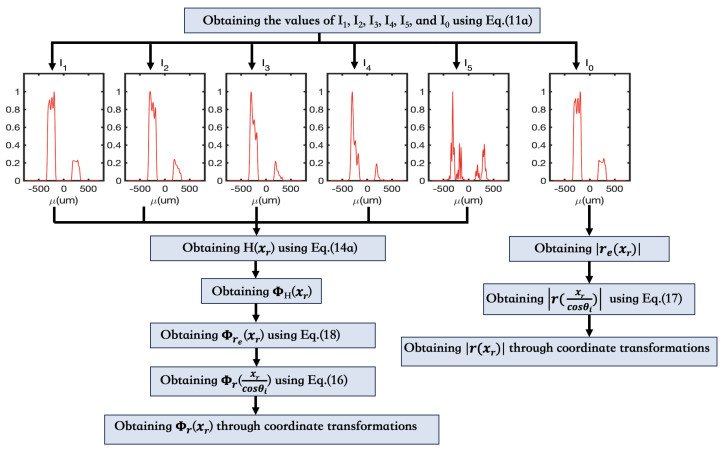
Flowchart of the FSPS Method for RGI: Here, we illustrate the application of an RDSGPP, generating curves $I_{1}\left(x_{r}\right)$ , $I_{2}\left(x_{r}\right)$ , $I_{3}\left(x_{r}\right)$ , $I_{4}\left(x_{r}\right)$ , $I_{5}\left(x_{r}\right)$ , and $I_{0}\left(x_{r}\right)$ as depicted in the figure. The specific parameters for this RDSGPP are $\left\{\rho_{1}=1,\;\rho_{2}=0.5,\tau_{1}=100\;\mu m,\tau_{2}=65\;\mu m\right\}$ .

These steps are further presented in the form of a flowchart in Figure 2.

**Figure 3:**
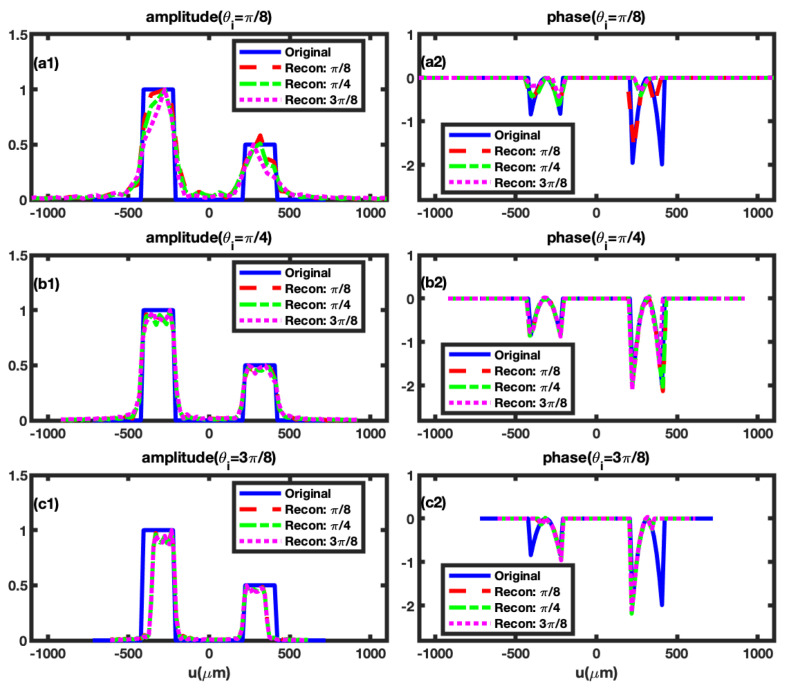
The obtained reflection coefficients for RDSGPPs are presented, considering different incident angles $\theta_{i}$ and various reflective angles $\theta_{o}$ . (**a1**,**a2**): With a fixed incident angle of $\theta_{i}=\pi /8$ , the reflective angles $\theta_{o}$ vary from π/8 to 3π/8 in intervals of π/8 . (**b1**,**b2**): Maintaining a constant incident angle of $\theta_{i}=\pi /4$ , the reflective angles $\theta_{o}$ span from π/8 to 3π/8 at π/8 intervals. (**c1**,**c2**): With the incident angle held at $\theta_{i}=3\pi /8$ , the reflective angles $\theta_{o}$ cover the range from π/8 to 3π/8 with intervals of π/8 .

To investigate the influence of different incident angles $\theta_{i}$ and reflective angles $\theta_{o}$ on our phase retrieval method, we exemplify using the RDSGPP and simulate the reconstructed results for nine distinct angle combinations presented in Figure 3. The reflection coefficient of the RDSGPP is defined as:

$$r_{1}\left(u\right)=\left\{\begin{array}{cc} \rho_{1}e^{-\frac{iu^{2}}{{\tau_{1}}^{2}}}, & \mathrm{if}\;-\frac{2w+d}{2}\leq u\leq -\frac{d}{2},\\ \rho_{2}e^{-\frac{iu^{2}}{{\tau_{2}}^{2}}}, & \mathrm{if}\;\frac{d}{2}\leq u\leq \frac{2w+d}{2},\\ 0, & \mathrm{other}. \end{array}\right.$$

Here, *w* denotes the slit width, and *d* represents the slit distance, both of which are set to 210μ m and 420μ m, respectively. Additionally, $\rho_{i}$ signifies the amplitude of the $i_{th}$ Gaussian plate slit, while $\tau_{i}$ denotes the width parameter governing the phase distribution for the $i_{th}$ Gaussian plate slit.

The parameters associated with the RDSGPP, as depicted in Figure 3, are $\rho_{1}=1,$ $\rho_{2}=0.5,\;\tau_{1}=100\;\mu m,\tau_{2}=65\;\mu m$ . In the figure, it is noticeable that when the incident angle $\theta_{i}$ is held constant, particularly in cases such as $\theta_{i}=\pi /4$ and $\theta_{i}=3\pi /8$ , the retrieval results exhibit only minor changes as the reflective angle $\theta_{o}$ varies between π/8 , π/4 , and 3π/8 , respectively. This observation suggests that the impact of the incident angle $\theta_{i}$ is considerably more significant than that of the reflective angle $\theta_{o}$ . Consequently, careful consideration is warranted when selecting the value of the incident angle $\theta_{i}$ . Although we have simulated only three incident angle cases, the results affirm that satisfactory retrieval outcomes are achieved when $\theta_{i}=\pi /4$ .

**Figure 4:**
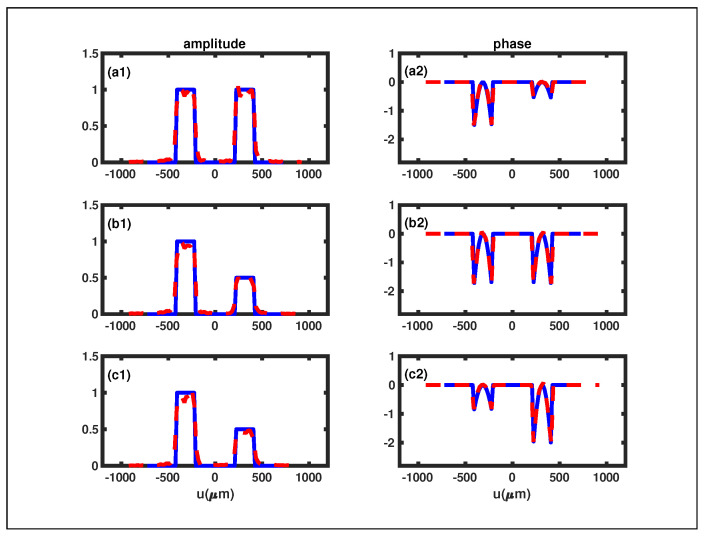
The retrieved reflection coefficients of three different RDSGPPs under the condition of $\theta_{i}=\theta_{o}=\pi /4$ . The original phases and amplitudes are depicted by the solid blue curves, whereas the reconstructed phases and amplitudes are illustrated by the dashed red curves. (**a1**,**a2**): $\rho_{1}=\rho_{2}=1,\tau_{1}=75\;\mu m,\tau_{2}=125\;\mu m$ . (**b1**,**b2**): $\rho_{1}=1,\rho_{2}=0.5,\tau_{1}=\tau_{2}=70\;\mu m$ . (**c1**,**c2**): $\rho_{1}=1,\rho_{2}=0.5,\tau_{1}=100\;\mu m,\tau_{2}=65\;\mu m$ .

Moreover, we note that when $\theta_{i}=\pi /4$ , the reconstruction result obtained with $\theta_{o}=\pi /4\left(\theta_{o}=\pi /8\right)$ surpasses that with $\theta_{o}=3\pi /8$ in Figure 3. Consequently, in subsequent simulations, we fix $\theta_{i}=\theta_{o}=\pi /4$ . Additionally, we simulate two other instances of RDSGPPs to further substantiate the reliability of the RGI phase retrieval method. The reconstructed phases and amplitudes of these plates are presented in Figure 4. The relative difference between the reconstructed phases and the initial phases is also close to zero, affirming the validity of our RGI phase retrieval scheme.

**Figure 5:**
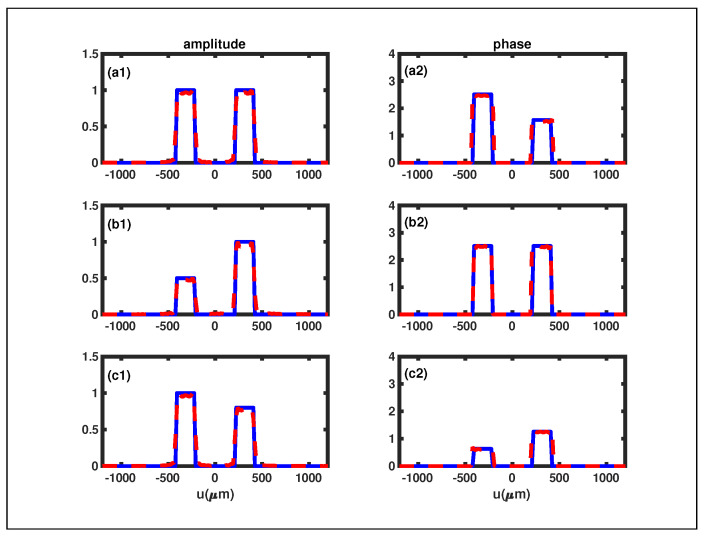
The retrieved reflection coefficients of the three different RDSs under the condition of $\theta_{i}=\theta_{o}=\pi /4$ . The original phases and amplitudes are depicted by the solid blue curves, whereas the reconstructed phases and amplitudes are illustrated by the dashed red curves. (**a1**,**a2**): $\psi_{1}=\psi_{2}=1$ , $\theta_{\psi_{1}}=0.8\pi ,\theta_{\psi_{2}}=0.5\pi$ . (**b1**,**b2**): $\psi_{1}=0.5,\psi_{2}=1$ , $\theta_{\psi_{1}}=\theta_{\psi_{2}}=0.8\pi$ . (**c1**,**c2**): $\psi_{1}=1,\psi_{2}=0.8$ , $\theta_{\psi_{1}}=0.2\pi ,\theta_{\psi_{2}}=0.4\pi$ .

The second example depicted in Figure 5 is the RDS with the reflection coefficient

$$r_{2}\left(u\right)=\left\{\begin{array}{ccc} \psi_{1}e^{i\theta_{\psi_{1}}} & \; & -\frac{2w+d}{2}\leq u\leq -\frac{d}{2}\\ \psi_{1}e^{i\theta_{\psi_{1}}} & \; & \frac{d}{2}\leq u\leq \frac{2w+d}{2}\\ 0 & \; & \mathrm{other}. \end{array}\right.$$

Here, $\psi_{i}$ and $\theta_{\psi_{i}}$ represent the amplitude and phase of the $i_{th}$ slit, respectively. To demonstrate the reliability of our RGI phase retrieval method, we selected three different RDSs, and their reconstructed phases and amplitudes are presented in Figure 5. In Figure 5, it is evident that the solid blue curves, representing the original phase and amplitude, closely align with the dashed red curves, representing the reconstructed phase and amplitude. This alignment indicates a substantial consistency between the reconstructed and original phases (amplitudes), providing additional evidence for the reliability of our phase reconstruction approach.

Finally, we would like to clarify why quantitative evaluation metrics, such as Signal-to-Noise Ratio (SNR), were not used in assessing the reconstruction results. The evaluation of SNR traditionally leans towards emphasizing amplitude information, making it a commonly used metric. SNR is conventionally computed by dividing the average signal value by the standard deviation of noise, with amplitude playing a pivotal role in this calculation. Phase information, on the other hand, is typically utilized to describe signal variations, periodicity, and relative positions, rather than directly influencing the signal’s intensity. Consequently, direct incorporation of phase information into SNR calculations is less prevalent, as phase is not inherently associated with the concept of ‘noise’.

It’s important to underscore that our amplitude reconstruction relies entirely on the established framework of the most traditional ghost imaging scheme. Additionally, we want to draw attention to our observation that the double integration function in MATLAB yielded suboptimal results when applied to integrate Equation (11a). This observation is manifested in the curve of $I_{0}$ depicted in Figure 2 (where $I_{0}$ is derived under the condition of $f(x_{1})=1$ , representing the outcome of the most traditional ghost imaging system). According to traditional ghost imaging theory, $I_{0}$ can be regarded as the square of the object’s amplitude. Ideally, if the double integration function in MATLAB were perfect, the curve of $I_{0}$ for each slit in Figure 2 should be parallel to the axis. However, it is evident that the curves of $I_{0}$ for each slit in Figure 2 do not align with the axis; instead, they exhibit significant fluctuations. This discrepancy indicates that the double integration function in MATLAB is not an ideal fit for simulating Equation (11a). Consequently, the reconstructed amplitude based on this foundation is also imperfect (e.g., the reconstructed amplitude in Figure 4 does not perfectly align with the original amplitude information). Similarly, we can infer that $I_{1}$ , $I_{2}$ , $I_{3}$ , $I_{4}$ , and $I_{5}$ obtained using this double integration function are not flawless, resulting in a deviation between the reconstructed phase information and theoretical values (as evident in simulation results, such as Figure 4).

Despite these challenges, it is crucial to highlight that even though $I_{0}$ , $I_{1}$ , $I_{2}$ , $I_{3}$ , $I_{4}$ , and $I_{5}$ obtained using the MATLAB double integration function are not perfect, and the simulation results (Figure 4 and Figure 5) maintain a high level of persuasiveness. This resilience indicates that the FSPS method remains a reliable approach.

## 4. Conclusions

In conclusion, through theoretical derivation and extensive simulations, we have demonstrated that the FSPS method can be directly applied to RGI for obtaining the phase distribution of reflecting objects. This method successfully reconstructed the reflection phases and amplitudes of three complex RDSGPPs and three complicated RDSs. We have explored different combinations of incident angles $\theta_{i}$ and reflective angles $\theta_{o}$ in simulations to analyze their effects on phase reconstruction performance. The simulation results reveal that the incident angle has a significantly greater impact than the reflective angle on phase retrieval results, emphasizing the importance of carefully choosing the incident angle. Our scheme exhibits robust performance when the incident angle is set to $\theta_{i}=\pi /4$ . Additionally, under the conditions of $\theta_{i}=\pi /4$ , the phase reconstruction result with $\theta_{o}=3\pi /8$ is less favorable than with $\theta_{o}=\pi /4$ ( $\theta_{o}=\pi /8$ ), suggesting a preference for smaller reflective angles ( $\theta_{o}\leq \pi /4$ ).

Crucially, the FSPS method sets itself apart from commonly used GI phase retrieval methods by eliminating the necessity for entangled photon pairs, avoiding the need for complex optical systems, skipping any Fourier transform steps, and foregoing lengthy iterative processes in its phase retrieval procedure. This distinction marks a departure from conventional GI phase retrieval methods. Furthermore, the FSPS method proves successful in acquiring object phase information, whether in TGI [34] or RGI, underscoring its remarkable versatility. We posit that this method is not only applicable to GI but also harbors substantial potential in various other phase retrieval applications.

## Data Availability

Data underlying the results presented in this paper are not publicly available at this time but may be obtained from the authors upon reasonable request.
